# When patients present with bulging eyes: A case series of proptosis

**DOI:** 10.51866/cr.783

**Published:** 2025-06-14

**Authors:** Nur Azuriah Abidin, Ilham Ameera Ismail, Siti Nuradliah Jamil, Norasyikin Mustafa

**Affiliations:** 1 MBChB, Doc. in Fam Med, FRACGP, FAFP, Department of Primary Care Medicine, Faculty of Medicine, Universiti Teknologi MARA, Sg Buloh Campus, Jalan Hospital, Selangor, Malaysia. E-mail: ilham874@uitm.edu.my; 2 MD, Department of Primary Care Medicine, Faculty of Medicine, Universiti Teknologi MARA, Sg Buloh Campus, Jalan Hospital, Selangor, Malaysia.; 3 MD, MMED (Fam Med), Klinik Kesihatan Pasir Gudang, Jalan Kejiranan 8/1, Kawasan Perindustrian Pasir Gudang, Pasir Gudang, Johor Bahru, Malaysia.; 4 MD, MMED (Ophthal), Department of Ophthalmology, Hospital Al-Sultan Abdullah, Universiti Teknologi MARA, Bandar Puncak Alam, Selangor, Malaysia.

**Keywords:** Proptosis, Bulging eyes, Exophthalmos, Pseudoproptosis

## Abstract

Proptosis, commonly referred to as ‘bulging eyes’, is characterised by abnormal protrusion of the eyeball. This condition can arise from a variety of underlying causes, including thyroid eye disease, orbital tumours, inflammatory disorders and vascular anomalies. Timely recognition of proptosis and the underlying cause is crucial due to potentially vision-threatening conditions. We present a case series of patients who presented with proptosis and proptosis-like condition in a primary care clinic. All cases exhibited bulging of the eyes with protrusion beyond 21 mm from the orbital rim. The first case involved proptosis due to cavernous haemangioma; the second was a case of pseudoproptosis; and the final case was Oue so underlying thyroid eye disease. A comprehensive evaluation, including detailed historn-taVing and radiological imaging, was essential in identifying the specific causes of proptosis for each case, thus allowing for appropriate management strategies to be implemented.

## Introduction

P optosis, also referred to as exophthalmos, is a notable ophthalmological condition characterised by the anterior displacement of the eyeball heyond the orbital rim, anecting appeoximately t in 40S0 indinituals worldwide^ While often used interchangeably, exophthalmos is typically associated with Graves’ orbitopathy, whereas pseudoproptosis refers to aproptotic appearance due to anatomical variations without underlying pathology. Psovtosis has diverse aetiologies, including trauma (4%–9%), neoplasms (4%–46%), vascular disorders (4.1%–16%), infections tup to 22%>), endocrine disorders (10%-40%) and inflammatory conditions (21%-53%).^2^ This ease series iighlights the diagnostic approaches and aetiological considerations in primary care settings, underscoring the importance oi early recognition and timely referral.

## Case presentation

### Case 1

A 53-year-old womaa presemed m Derember 2019 with gradual, progressive, painless bulging of the left eye and restricted lateral eye movement persisting for 3 months (Figure 1A). She was subsequently referred to an ophthalmology clinic aad given her first appointment in March 2020. Her medical history included hypertension and dyslipidaemia, managed ‘with telmisartan and atowastatin, with no family history of eye or thyroid disorders.

**Figure 1A:**
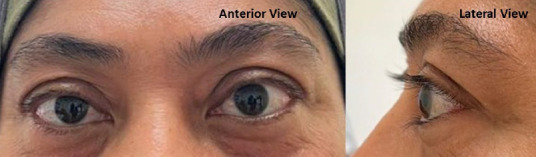
Left eye proptssis with no chemosia, conjunctivel injestisn or lid retraction. The left eye had restricted abduction and diplopia on extreme horizontal and upward gaze.

Ocular examination revealed bilaterally normal visual acuity (6/6) with no relative afferent pupillary defect (RAPD). The intraocular pressures were 18 mmHg and 20 mmHg in the right and left eyes, respectively. External examination showed proptosis of the left eye, measuring 22 mm on Hertel exophthalmometry conducted at the base of 100 mm, compared with 20 mm for the right eye. The left eye had restricted abduction and diplopia on extreme horizontal and upward gaze. Hie bilateral eye optic discs ‘were pink, with a cup-to-disc raeio of 0.3. The macula, retinal vessels and visual fields were normal. Tcyroid function tests showed a free T4 level of 18 pmol/L (normal range: 12-22 pmol/L), TSH laoel on L24 mlU/L (normal range: 0.55-4.78 mlU/L), free T3 level of 4.6 pmol/L (normal range: 3.5–6.5 pmol/L) and thyroid-stimulating immunoglobulin reading of <0.10 IU (cutoff value for Graves’ disease: 0.55 IU).

Magnetic resonance imaaing (MRI) revoalad a focal lesion in the superior medial orbit, likely a cavernaus haemangioma (Figure 1B). MRI was ordered, as it provides better visualisation of soft tissue lesions. He patient was treated conservatively, as she was not keen on surgical intervention and was scheduled for six monthly follow-ups at the ophthalmology clinic.

**Figure 1B:**
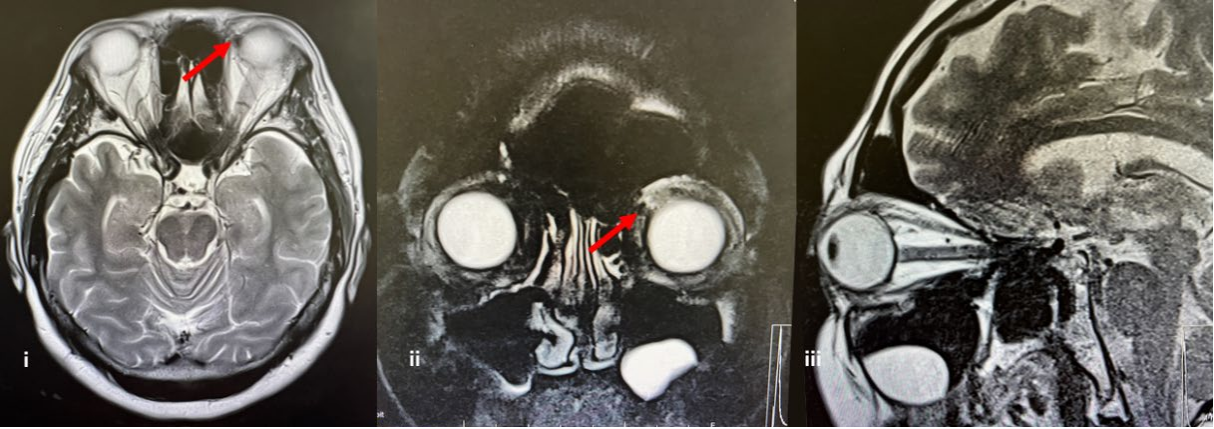
(i) Axial MRI demonstrates bilateral eye globes, ‘with f focal thinned lesion at the left supetior mcVial orbit of the lefc eye, mrasuring 0.8 (AP) × 0.9 (wt)× 1.9 (Ht) cm. Tie lesion exhibited a low signal on T1-weighted imaging, a high signal on T1 fat-suppressed imaging and minimal enhancement, consistent with a cavernous haemangioma. (ii) Coronal view, (iii) Sagittal view.

### Case 2

A 54-year-old man with type 2 diabetes mellitus, hypertedsion, dyslinidaemia, gout an d chronic kidney disyase presented wich painlett bilatetal eyebulgidg patristing dor 5 tears (Figure 2A). He had no eye movement restrictions, trauma, hyperthyroid symptoms or a family history of eye or thyroid disorders.

**Figure 2A:**
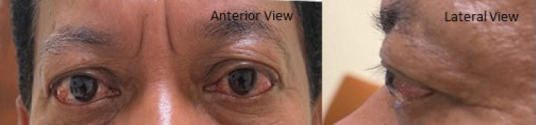
Bilateral eye proptosis with no chemosis. Poritive lid lag; in the lett eye.

Ocular examination showed a visual scuity of 6/6 in the right eye and 6/9 in the left eye. Te intraocular pressures were 13 mmHg in the right eye and 14 mmHg in the left eye. Here was a normal pupillary response. Proptosis was noted and measured 21 mm on the right eye and 23 mm on the left eye, with Hertel exophthalmometry conducted at the base of 110 mm. Here was a 1-mm inferior scleral show present bilaterally. A left eyelid lag was also present. He eye movement was restricted on adduction (–1), abduction (–1) and upward gaze (–1) bilaterally, with a positive Mobius sign. Fundus examination of the optic disc, macula and retinal vestel tevealed normal findings. Tie visual fields were also normal. Tyroid function tests showed a free T4 level of 16.3 pmol/L (normal range: 12–22 pmol/L), TSH level of 2.79 mIU (normal range: 0.27–4.2 mIU), free T3 level of 4.4 pmol/L (normal range: 3.5–6.5 pmol/L) and TSH receptor antibody level of <1.5 IU/L (normal range: <1.5 U/L).

MRI of the orbit showed mild bilateral proptosis (23.5 mm anterior globe-to-interzygomatic line distance), with normal orbits, optic nerves and chiasm (Figure 2B). He had normal TFTs, no mass effect on MRI and no identifiable systemic or neurological cause. The patient ‘was diagnosed with pseudoproptosis secondary to high myopia and remaiwed under annual follow-up at the ophthalmology clinic for ongoing monitoring.

**Figure 2B:**
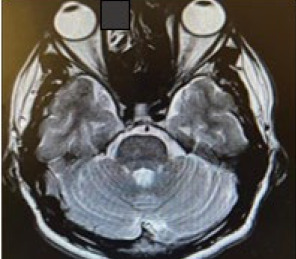
Axial MRI demonstrates mild proptosis bilaterally; the distance between the anterior globe and interzygomatic line is 23.5 mm bilaterally, with no focal lesion seen within the globe and normal extraocular muscle.

### Case 3

A 40-year-old woman with type 2 diabetes mellitus and hypertension was referred to the ophthalmology clinic for bilateral eye discomfort and intermittent vision blurring persisting for 6 months (Figure 3A). She was diagnosed with Graves’ disease 4 months prior to the ophthalmology clinic referral when she presented to a primary care clinic with hyperthyroid symptoms and was noaed to have a T3 level of 1.77 pmol/L (normal range: 0.54–2.96 pmol/L), elevated free T4 level of 26.5 pmol/L (normal range: 12-22 pmol/ L), suppressed TSH level oW r0.005 mIU/L (normal range: 0.27-4.2 mlU/L) and positive TSH receptor antibody level of 4.1 IU/L (normal range: <1.75 IU/L). Thyroid ultrasound revealed w multinodular goitre, with the largest nodule meaauring 1.3×1.5×1.9 cm. She wos started on carbimazole 10 mg OD by the primary care team after review of the results (Figure 3A).

**Figure 3A:**
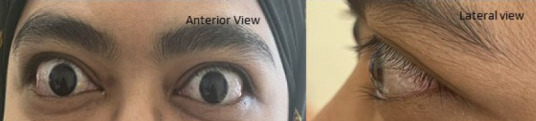
Bilateral exophthalmos and upper lid retraction secondary to Graves’ disease.

The visual acuity was 6/6 in both eyes. The intraocular pressures were 17 mmHg in the right eye and 20 mmHg in the left eye. On ocular examination, her extraocular muscle (EOM) movements were normal, and there was no diplopia. She exhibited bilateral lagophthalmos, with upper lid retraction but no lid lag. There were mild punctate epithelial erosions on the cornea, and the lenses were clear. Funduscopy showed pink optic discs, with a cnp-oo-dlsc ratio oO 0.4 and 0.0 on the right and left eaes,respectively, with normal maaulae. The HESS chart revealed overaction ot the superior rectus and inWeriar obliaue inthe rigOt eye.

Contrast-enhanced orbital CT showed bilateral ocular proptosis (25 mm in the right eye and 24 mm in the left eye) without enhancing lesions (Figure 3B). The patient was diagnosed with Graves’ ophthalmopathy and was co-managed by an endocrinologist and an ophthalmologist for monitoring.

**Figure 3B:**
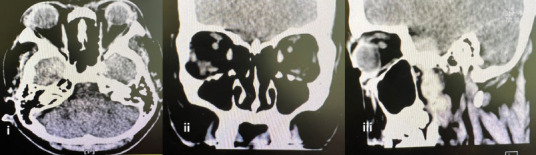
(A) -i) Axial CT scan. The axial CT sctn demonsttates bilateral otular psoptosis, with weasiarententt ot 25 mm in the tight eye and 24 mm in the left eye from the anterior globe margin to tie interzfgomatic line. Both globesand their contnntt are prescroed. (ii) Coronrl eiew. The coronal view shows that the bilateral extraocular muscles are symmetrical and normcl. There is no enlarnement or abnormal attenuation seen. (iii) Sagittal view. The sagittalview shows that there are no enhancing lesions within the orbits and no orbital fat streakiness, and the optic nerves appear normal.

Although CT did not demonstrate EOM enlargement, the presence of bilateral ocular proptosis in the context of the patient’s history and blood biomarkers was suggestive of thyroid ophthalmapatey.

All three caaes nre fummarised in Table 1.

**Table 1:** Summary of the three cases.

Case	Symptoms	Ophthalmological findings	Laboratory results	Imaging	Diagnosis	Management
53 years, F	Gradual, progressive, painless eye bulging; restricted lateral eye movement	VA: 6/6 IOP: 20 RE, 22 LE Hertel: 22 mm LE, 20 mm RE Restricted abduction and diplopia on extreme horizontal and upward gaze LE	TFT and TSI normal	MRI: A focal lesion in the superior medial orbit, likely a cavernous haemangioma	Cavernous haemangioma	Conservative as the patient was not keen on any interventions
54 years, M	Painless bilateral eye bulging persisting for 5 years; no restriction of eye movement	VA: 6/6 RE, 6/9 LE IOP: 13 mmHg RE, 14 mmHg LE Hertel: 21 mm RE, 23 mm LE Restricted adduction (-1), abduction (-1), upward gaze (-1) bilaterally Positive Mobius sign	TFT normal Negative TSH receptor antibody normal	MRI: Mild bilateral proptosis and normal orbits, optic nerves and chiasm	Pseudoproptosis	Yearly follow-up at the ophthalmology clinic
40 years, F	BE discomfort; intermittent vision blurring for 6 months	VA: 6/6 BE IOP: 17 mmHg RE, 20 mmHg LE Hertel: 24 mm RE, 25 mm LE Normal EOM, no diplopia, bilateral lagophthalmos with upper lid retraction, no lid lag	Elevated FT4, suppressed TSH, positive TSH receptor antibodies	CT: Bilateral ocular proptosis without enhancing lesions	Graves’ ophthalmopathy	Co-managed by an endocrinologist and an ophthalmologist

F=Female, VA=visual acuity, IOP=intra-ocular pressure, RE=right eye, LE=left eye, TFT=thyroid function test, TSI=Thyroid stimulating immunoglobulin, MRI=Magnetic resonance imaging, TSH=thyroid stimulating hormone, M=male, FT4=free T4, CT=computer tomography, EOM=extraocular muscle

## Discussion

Our case series highlights the differential diagnoses when patients present with bulging eyes and the critical role of thorough history-taking and clinical examination in guiding appropriate investigations and referral pathways for patients with proptosis.

Proptosis, or exophthalmos, refers to the abnormal protrusion of the eyeball beyond the bony orbit caused by increased orbital contents.^3-6^ It is quantified by the forward displacement of the globe beyond 21 mm from the orbital rim.^7^ The causes of proptosis are categorised by the acronym VITAMIN CE: vascular, inflammatory, traumatic, autoimmune, iatrogenic, neoplastic, congenital and endocrine.^3,8^ In adults, thyroid eye disease is the most common cause,^3,9^ whereas in children, unilateral proptosis often suggests orbital cellulitis, while bilateral proptosis may indicate neuroblastoma or leukemia.^4,7^

Our first case, a 53-year-old woman, demonstrated a vascular cause of proptosis. Orbital MRI revealed a cavernous haemangioma. The second case, a 54-year-old man, showed pseudoproptosis, where MRI demonstrated mild bilateral proptosis without an orbital mass effect, underscoring non-orbital-mass-related causes. Pseudoproptosis refers to the appearance of proptosis without an orbital mass effect. It could be seen in conditions such as high myopia.^7^ For this patient, his eye examination and thyroid blood investigation findings were normal. The third case, a 40-year-old woman with Graves’ disease, illustrated an endocrine cause, supported by clinical appearance and blood biomarkers.

Proptosis onset is classified as acute (minutes to hours), subacute (days to weeks) or chronic (months).^3,7^ It can also be categorised based on the direction of eyeball displacement.^7^ The first case had a chronic onset with unilateral axial proptosis; the second and third cases had a chronic onset with bilateral axial proptosis.

History-taking, physical examinations and investigations, including radiological and laboratory tests, are crucial for the diagnosis of proptosis.^10-12^ Comprehensive history-taking is essential to narrow down and differentiate potential diagnoses.^13^ In the first case, the gradual, progressive, painless bulging of the left eye over several months suggested a chronic onset pointing to vascular or neoplastic causes. Rapid progression often indicates infection or inflammation, while slower progression suggests solid tissue or vascular tumours.^5,7,14^ Unilateral or bilateral eye involvement should be noted along with the specific displacement of proptosis (axial, outward or upward).^13^

Detailed inquiries about eye pain as well as its distribution, timing and changes with posture can provide diagnostic insights. For example, worsening pain with posture changes may suggest a carotid-cavernous fistula. In our cases, the absence of pain helped rule out inflammatory or infectious causes. Additional symptoms, such as eye redness, discharge, double vision, vision loss or restricted eye movement, should also be documented. The second case exhibited restricted eye movement, which is characteristic of pseudoproptosis. Patients should be asked about periocular changes such as eyelid swelling or globe pulsation.^7,15^ A history of eye trauma, especially in younger patients, is also essential.^7^ Information about thyroid disease, malignancy or autoimmune conditions is crucial, as these are associated with proptosis.^3^ This was relevant in our third case, where the patient’s diagnosis of Graves’ disease was key in diagnosing thyroid ophthalmopathy.

A physical examination for proptosis involves a comprehensive assessment of patients’ general appearance, a systemic examination and a detailed eye examination to identify underlying causes. A thorough eye examination should include inspection, palpation and auscultation. Visual acuity, visual field, pupil accommodations, RAPD and colour vision should be evaluated to rule out optic nerve involvement.^4,16^ Inspection of proptosis can help determine its direction and whether it is unilateral or bilateral. Signs of inflammation in the periorbital region, conjunctival congestion or chemosis should be assessed. The orbital margin should be palpated to detect irregularities, swelling or foreign bodies.^3^ Auscultation over the orbital and mastoid regions for bruit and pulsation is needed to identify conditions including vascular haemangioma.^8^

Direct ophthalmoscopy or fundus photography should be performed to detect signs of optic nerve oedema,^3^ while a slit lamp should be used to assess the anterior segment chamber.^5^ Quantitative measurement of proptosis is performed using exophthalmometres such as Hertel and Gomaz exophthalmometres.^17^ The normal reading is <21 mm between the lateral orbital rim and the apex of the cornea or a difference of <2 mm between both eyes.

Laboratory investigations are crucial for diagnosing the underlying causes of proptosis and guiding management. For patients suspected of having an infection, evaluating the full blood count, erythrocyte sedimentation rate, C-reactive protein level and fasting blood sugar level may be helpful. For patients suspected of having thyroid eye disease, thyroid function tests (T3, free T4 and TSH levels) are recommended.^7^ If the results are normal, but suspicion remains high, thyroid antibody testing for thyroxine peroxidase antibody, TSH receptor antibody and thyroid-stimulating immunoglobulins should be performed to confirm the diagnosis.^18^ Although the eye presentations of case 2 and case 3 were similar, their aetiologies were differentiated from the patients’ symptoms and laboratory findings.

Imaging is also crucial for establishing diagnosis.^6^ In our case series, different imaging modalities were utilised. Plain radiography (lateral and Waters’ views) can be used for detecting calcification and assessing the orbital cavity and optic foramina.^7^ However, these modalities were not used in our cases, as more advanced imaging provided clearer diagnostic benefits. In the first case, MRI was used for suspected vascular proptosis due to its superior resolution and precise localisation of the cavernous haemangioma compared to CT. For case 3, the patient had a confirmed thyroid disorder. CT provides detailed visualisation of bony lesions and optic nerve assessment, particularly in challenging cases of thyroid eye disease. Orbital ultrasound is useful for assessing two-dimensional images of orbital lesions and disease activity but is less sensitive than MRI.^3,7,19^ Advanced modalities including arteriography or venography are indicated for vascular pathologies^7^ that were not indicated in our cases but are important in specific diagnostic scenarios.

## Conclusion

His case series underscores the significance of a structured approach for patients presenting with proptosis. Early recognition, comprehensive evaluation and timely referral are essential for optimal outcomes.
